# Gene annotation and network inference by phylogenetic profiling

**DOI:** 10.1186/1471-2105-7-80

**Published:** 2006-02-17

**Authors:** Jie Wu, Zhenjun Hu, Charles DeLisi

**Affiliations:** 1 Department of Biomedical Engineering, Boston University, 24 Cummington St., Boston, MA, 02215, USA; 2 Bioinformatics and Systems Biology, Boston University, 24 Cummington St., Boston, MA, 02215, USA

## Abstract

**Background:**

Phylogenetic analysis is emerging as one of the most informative computational methods for the annotation of genes and identification of evolutionary modules of functionally related genes. The effectiveness with which phylogenetic profiles can be utilized to assign genes to pathways depends on an appropriate measure of correlation between gene profiles, and an effective decision rule to use the correlate. Current methods, though useful, perform at a level well below what is possible, largely because performance of the latter deteriorates rapidly as coverage increases.

**Results:**

We introduce, test and apply a new decision rule, correlation enrichment (CE), for assigning genes to functional categories at various levels of resolution. Among the results are: (1) CE performs better than standard guilt by association (SGA, assignment to a functional category when a simple correlate exceeds a pre-specified threshold) irrespective of the number of genes assigned (*i.e*. *coverage*); improvement is greatest at high coverage where precision (positive predictive value) of CE is approximately 6-fold higher than that of SGA. (2) CE is estimated to allocate each of the 2918 unannotated orthologs to KEGG pathways with an average precision of 49% (approximately 7-fold higher than SGA) (3) An estimated 94% of the 1846 unannotated orthologs in the COG ontology can be assigned a function with an average precision of 0.4 or greater. (4) Dozens of functional and evolutionarily conserved cliques or quasi-cliques can be identified, many having previously unannotated genes.

**Conclusion:**

The method serves as a general computational tool for annotating large numbers of unknown genes, uncovering evolutionary and functional modules. It appears to perform substantially better than extant stand alone high throughout methods.

## Background

One of the remarkable characteristics of the genomic era is that the solution to the challenge of annotation posed by the rapid increase in sequences, comes in part from the data itself; *i.e*. the availability of a large number of fully sequenced genomes provides information that enables the development of new computational approaches including domain fusion [1-3], chromosomal proximity [4] and phylogenetic profiling [5-8].

Phylogenetic profiling, in its original form, was used to infer the function of a gene by finding another gene of known function with an identical pattern of presence and absence across a set of phylogenetically distributed genomes. Such restricted profiling, requiring full profile identity, while accurate, has low coverage, assigning pathways to 114 of 1814 unknown orthologous proteins from 44 genomes [9], with an estimated accuracy in the vicinity of 90%. The restriction can be relaxed in a number of ways, using a Pearson correlation, Mutual information [6,8,9], or mathematically exact statistical significance assignment. In a previous paper [9] we examined each of these methods, and settled on the last of them as a convenient and generally valid measure.

Briefly, the phylogenetic profile of a gene is a binary string recording the presence (1) or absence (0) of an ortholog across a suitable set of genomes. We use orthologs as defined in the COG database [10,11]. If the correlation between the profiles of two genes, *X *and *Y*, is much greater than would be expected by chance, then they are assumed to be functionally related. Let *N *be the number of genomes over which the profiles are defined, with gene *X *occurring in *x *genomes, *Y *occurring in *y *genomes, and both occurring in *z *genomes. Assuming the gene content of all genomes are independent of each other, then *P*(*z *| *N*, *x*, *y*), the probability of observing *z *co-occurrences purely by chance, given *N*, *x *and *y *is

P(z|N,x,y)=(N−xy−z)(xz)(Ny)=(N−x)!(N−y)!x!y!(N+z−x−y)!(x−z)!(y−z)!z!N!     (1)
 MathType@MTEF@5@5@+=feaafiart1ev1aaatCvAUfKttLearuWrP9MDH5MBPbIqV92AaeXatLxBI9gBaebbnrfifHhDYfgasaacH8akY=wiFfYdH8Gipec8Eeeu0xXdbba9frFj0=OqFfea0dXdd9vqai=hGuQ8kuc9pgc9s8qqaq=dirpe0xb9q8qiLsFr0=vr0=vr0dc8meaabaqaciaacaGaaeqabaqabeGadaaakeaacqWGqbaucqGGOaakcqWG6bGEcqGG8baFcqWGobGtcqGGSaalcqWG4baEcqGGSaalcqWG5bqEcqGGPaqkcqGH9aqpdaWcaaqaamaabmaaeaqabeaacqWGobGtcqGHsislcqWG4baEaeaacqWG5bqEcqGHsislcqWG6bGEaaGaayjkaiaawMcaamaabmaaeaqabeaacqWG4baEaeaacqWG6bGEaaGaayjkaiaawMcaaaqaamaabmaaeaqabeaacqWGobGtaeaacqWG5bqEaaGaayjkaiaawMcaaaaacqGH9aqpdaWcaaqaaiabcIcaOiabd6eaojabgkHiTiabdIha4jabcMcaPiabcgcaHiabcIcaOiabd6eaojabgkHiTiabdMha5jabcMcaPiabcgcaHiabdIha4jabcgcaHiabdMha5jabcgcaHaqaaiabcIcaOiabd6eaojabgUcaRiabdQha6jabgkHiTiabdIha4jabgkHiTiabdMha5jabcMcaPiabcgcaHiabcIcaOiabdIha4jabgkHiTiabdQha6jabcMcaPiabcgcaHiabcIcaOiabdMha5jabgkHiTiabdQha6jabcMcaPiabcgcaHiabdQha6jabcgcaHiabd6eaojabcgcaHaaacaWLjaGaaCzcamaabmaabaGaeGymaedacaGLOaGaayzkaaaaaa@7C58@

The connection between equation (1) and the more readily calculated mutual information, *MI*(*X*, *Y*), of the profile pair, is easily if tediously established. In particular for a given profile pair, define *p*(*i*, *j*), (*i *= 0, 1; *j *= 0, 1) as the fraction of genomes in which gene *X *is in state *i*; *i.e*. present (*i *= 1), or absent (*i *= 0), and gene *Y *is in state *j*, so that *p*(1, 1) is the fraction of genomes in which both genes are present, *p*(1, 0) is the fraction in which *X *is present and *Y *is absent, *etc*. In addition p(i)=∑j=01p(i,j)
 MathType@MTEF@5@5@+=feaafiart1ev1aaatCvAUfKttLearuWrP9MDH5MBPbIqV92AaeXatLxBI9gBaebbnrfifHhDYfgasaacH8akY=wiFfYdH8Gipec8Eeeu0xXdbba9frFj0=OqFfea0dXdd9vqai=hGuQ8kuc9pgc9s8qqaq=dirpe0xb9q8qiLsFr0=vr0=vr0dc8meaabaqaciaacaGaaeqabaqabeGadaaakeaacqWGWbaCcqGGOaakcqWGPbqAcqGGPaqkcqGH9aqpdaaeWbqaaiabdchaWjabcIcaOiabdMgaPjabcYcaSiabdQgaQjabcMcaPaWcbaGaemOAaOMaeyypa0JaeGimaadabaGaeGymaedaniabggHiLdaaaa@3F5E@ and p(j)=∑i=01p(i,j)
 MathType@MTEF@5@5@+=feaafiart1ev1aaatCvAUfKttLearuWrP9MDH5MBPbIqV92AaeXatLxBI9gBaebbnrfifHhDYfgasaacH8akY=wiFfYdH8Gipec8Eeeu0xXdbba9frFj0=OqFfea0dXdd9vqai=hGuQ8kuc9pgc9s8qqaq=dirpe0xb9q8qiLsFr0=vr0=vr0dc8meaabaqaciaacaGaaeqabaqabeGadaaakeaacqWGWbaCcqGGOaakcqWGQbGAcqGGPaqkcqGH9aqpdaaeWbqaaiabdchaWjabcIcaOiabdMgaPjabcYcaSiabdQgaQjabcMcaPaWcbaGaemyAaKMaeyypa0JaeGimaadabaGaeGymaedaniabggHiLdaaaa@3F5E@. Then the relation between equation (1) and the mutual information

MI(X,Y)≡−∑i=01∑j=01p(i,j)log⁡p(i,j)p(i)p(j)     (2)
 MathType@MTEF@5@5@+=feaafiart1ev1aaatCvAUfKttLearuWrP9MDH5MBPbIqV92AaeXatLxBI9gBaebbnrfifHhDYfgasaacH8akY=wiFfYdH8Gipec8Eeeu0xXdbba9frFj0=OqFfea0dXdd9vqai=hGuQ8kuc9pgc9s8qqaq=dirpe0xb9q8qiLsFr0=vr0=vr0dc8meaabaqaciaacaGaaeqabaqabeGadaaakeaacqWGnbqtcqWGjbqscqGGOaakcqWGybawcqGGSaalcqWGzbqwcqGGPaqkcqGHHjIUcqGHsisldaaeWbqaamaaqahabaGaemiCaaNaeiikaGIaemyAaKMaeiilaWIaemOAaOMaeiykaKcaleaacqqGQbGAcqGH9aqpcqaIWaamaeaacqaIXaqma0GaeyyeIuoaaSqaaiabdMgaPjabg2da9iabicdaWaqaaiabigdaXaqdcqGHris5aOGagiiBaWMaei4Ba8Maei4zaC2aaSaaaeaacqWGWbaCcqGGOaakcqWGPbqAcqGGSaalcqWGQbGAcqGGPaqkaeaacqWGWbaCcqGGOaakcqWGPbqAcqGGPaqkcqWGWbaCcqGGOaakcqWGQbGAcqGGPaqkaaGaaCzcaiaaxMaadaqadaqaaiabikdaYaGaayjkaiaawMcaaaaa@61F3@

is [12]:

MI(X,Y)=−limN→∞1Nlog⁡2P(z|N,x,y)     (3a)
 MathType@MTEF@5@5@+=feaafiart1ev1aaatCvAUfKttLearuWrP9MDH5MBPbIqV92AaeXatLxBI9gBaebbnrfifHhDYfgasaacH8akY=wiFfYdH8Gipec8Eeeu0xXdbba9frFj0=OqFfea0dXdd9vqai=hGuQ8kuc9pgc9s8qqaq=dirpe0xb9q8qiLsFr0=vr0=vr0dc8meaabaqaciaacaGaaeqabaqabeGadaaakeaacqWGnbqtcqWGjbqscqGGOaakcqWGybawcqGGSaalcqWGzbqwcqGGPaqkcqGH9aqpdaWfqaqaaiabgkHiTGqaciab=XgaSjab=LgaPjab=1gaTbWcbaGaemOta4KaeyOKH4QaeyOhIukabeaakmaalaaabaGaeGymaedabaGaemOta4eaaiGbcYgaSjabc+gaVjabcEgaNnaaBaaaleaacqaIYaGmaeqaaOGaemiuaaLaeiikaGIaemOEaONaeiiFaWNaemOta4KaeiilaWIaemiEaGNaeiilaWIaemyEaKNaeiykaKIaaCzcaiaaxMaadaqadaqaaiabbodaZiabbggaHbGaayjkaiaawMcaaaaa@56E6@

In this paper we therefore define a new and fully general measure of correlation between two binary strings

C(z|N,x,y)≡−1Nlog⁡2P(z|N,x,y)0≤C≤1     (3b)
 MathType@MTEF@5@5@+=feaafiart1ev1aaatCvAUfKttLearuWrP9MDH5MBPbIqV92AaeXatLxBI9gBaebbnrfifHhDYfgasaacH8akY=wiFfYdH8Gipec8Eeeu0xXdbba9frFj0=OqFfea0dXdd9vqai=hGuQ8kuc9pgc9s8qqaq=dirpe0xb9q8qiLsFr0=vr0=vr0dc8meaabaqaciaacaGaaeqabaqabeGadaaakeaafaqabeqacaaabaGaem4qamKaeiikaGIaemOEaONaeiiFaWNaemOta4KaeiilaWIaemiEaGNaeiilaWIaemyEaKNaeiykaKIaeyyyIORaeyOeI0YaaSaaaeaacqaIXaqmaeaacqWGobGtaaGagiiBaWMaei4Ba8Maei4zaC2aaSbaaSqaaiabikdaYaqabaGccqWGqbaucqGGOaakcqWG6bGEcqGG8baFcqWGobGtcqGGSaalcqWG4baEcqGGSaalcqWG5bqEcqGGPaqkaeaacqaIWaamcqGHKjYOcqWGdbWqcqGHKjYOcqaIXaqmaaGaaCzcaiaaxMaadaqadaqaaiabbodaZiabbkgaIbGaayjkaiaawMcaaaaa@597E@ 0 ≤ *C *≤ 1 (3b)

As a rule of thumb, the difference between *MI *and the more general correlate, eq 3b, can safely be ignored for profiles when all variables are greater than 10. In this paper we expect only inconsequential differences between eqs 2 and 3b since we will be looking at profiles across 66 microbes (in contrast to looking only at eukaryotes or only archaea).

The simplest decision rule on which to base the correlate is *guilt by association (SGA) *[13-15], which assigns an unannotated gene to all known categories of an annotated gene if the phylogenetic profiles exceed some specific *correlation threshold*, C*. Assessments of this procedure often look promising. For example, a threshold of C^* ^= 0.35 (p^* ^= 10^-7^), links 1025 of the 2,918 unannotated orthologs to at least one pathway annotated gene, and 80% (820) are estimated to be correctly linked at least once. As we indicate below, however, such an assessment criterion conveys an overly optimistic picture of performance.

In contrast to *SGA*, *Correlation enrichment (CE) *assigns an unannotated gene by ranking each category (pathway) with a score reflecting (i) the *number *of (annotated) genes within a category, whose profile correlation with that of the unannotated gene exceeds a pre-specified threshold, and (ii) the magnitudes of these correlations (see materials and methods)

One of the difficulties in comparing different methods is a lack of standardized performance measures. Different authors sometimes use different measures of performance (see for example [15-17]); performance is not always fully assessed; the same measure is sometimes defined in different ways, and performance as a function of coverage is not always available. In this paper we therefore evaluate a complete set of performance measures and their response characteristics as coverage is varied, against three different ontologies. We find that *CE *substantially outperforms *SGA *in allocating genes to functional categories. We were able to assign all 2918 KEGG unannotated orthologs to pathways with an estimated average precision of 49%, and all COG unannotated orthologs to COG categories, with an estimate of precision for each assignment. Finally, we identify several dozen cliques or quasi-cliques, some only partially annotated, placing unannotated genes in evolutionarily conserved functional modules with very high reliability.

## Results and Discussion

### Comparison of decision rules

**Figure 1:**
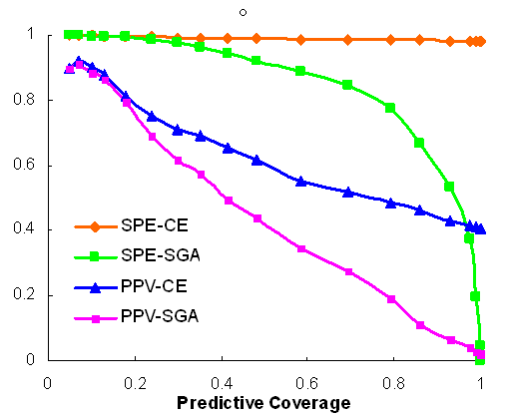
Specificity and positive predictive value as a function of predictive coverage for SGA and CE decision rules. Coverage is a function of correlation threshold, C*.

The simplest embodiment of *SGA *is assignment based on profile identity [18]. For pathway inferences based on identity, all measures of reliability are very high (Table 1), but only 5.4% of unannotated orthologs are assignable to KEGG pathways Relaxing the requirement for an exact match increases coverage and the expected number of correct predictions, but specificity and positive predictive value (PPV) both deteriorate markedly (Figure 1). For example setting correlation threshold C* = 0.2 (p* = 10^-4^) to achieve a coverage of 90% requires accepting a *PPV *of 6%. Notably, although *PPV *is very low at C* = 0.2, 90% of the genes are assigned correctly to at least one pathway, indicating that *A*_0 _(the fraction of genes assigned correctly to at least one pathway) is not a useful measure of performance. When inferences are based on correlation enrichment, *PPV *is markedly increased at high coverage, exceeding its *SGA *value approximately 6 fold, whereas the two decision rules perform similarly at coverages below 20% (Figure 1).

**Table 1:** Pathway allocation performance of using exactly matching phylogenetic profiles. AA (UU) denotes pairs in which both genes are annotated (unannotated), and AU denotes pairs with one annotated and one unannotated gene. N is the number of links. G is the number of genes that form those links (unannotated genes in AU). N^* ^is the number of links between genes that share at least one path; G^* ^is the number of such genes. PPV, A_0_, A_C_, sensitivity and specificity are defined as in Material and Methods.

	**N**	N^*^	**G**	G^*^	PPV¯ MathType@MTEF@5@5@+=feaafiart1ev1aaatCvAUfKttLearuWrP9MDH5MBPbIqV92AaeXatLxBI9gBaebbnrfifHhDYfgasaacH8akY=wiFfYdH8Gipec8Eeeu0xXdbba9frFj0=OqFfea0dXdd9vqai=hGuQ8kuc9pgc9s8qqaq=dirpe0xb9q8qiLsFr0=vr0=vr0dc8meaabaqaciaacaGaaeqabaqabeGadaaakeaadaqdaaqaaiabdcfaqjabdcfaqjabdAfawbaaaaa@3044@	**A**_0_	**A**_C_	**SEN**	**SPC**
AA	288	254	249	234	85%	94%	90%	91%	99%
AU	271	(239)	159	(149)					
UU	1090	NA	603	NA					

*PPV *estimates are conservative: assignment of a gene to a pathway in which it is currently not annotated could mean that the presence of the gene in that pathway has not yet been discovered; *i.e*. such assignments need not be false positives, even though they are counted as such. That many of the putative false positives are in fact functionally related to the assigned pathway is seen by searching the *GO *ontology. In particular, of the 602 genes that are allocated to *KEGG *pathways in which they are currently not annotated (*FP*), 467 have *GO *annotations. Of those 467, more than 60% share at least one *GO *category at a depth of 5 or greater, with the pathway genes. The fact that an unannotated gene shares a *GO *category with genes in the pathways to which it is assigned suggests that these are plausible predictions rather than false positives.

**Figure 2:**
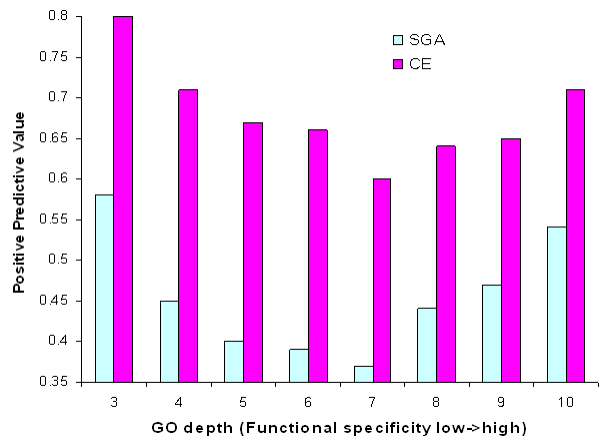
*PPV *as a function of *GO *depth for *CE *and *SGA *decision rules, using a correlation threshold of C* = 0.40 (p* = 10^-8^). The predictive coverage is approximately 25%.

A more general assessment against the *Gene Ontology *confirms the superior performance of *CE*. For example, at C* = 0.40 where the *SGA *and *CE *curves for positive predictive value have reached about half their maximum divergence (Figure 1), *CE *performs substantially better than *SGA *at all *GO *specificity levels (Figure 2). The use of *MI *(eq 2) rather than the more general relation (eq 3b) has essentially no effect on these results (data not shown).

### Comparison with other published methods

Non-homology based functional assignments have been made using a number of different datasets, including evolutionary methods, expression profiling [19,20], large scale protein-protein interaction (*PPI*) data [21], microRNA targeted mRNA [22] and pattern of annotation [23]. For example the function of an annotated gene is transferred to an unannotated gene if they are found to interact via the yeast two hybrid assay, or if the correlation in their expression profiles exceed a fixed, arbitrarily set threshold. For any given dataset, a number of different methods have been proposed to draw functional inferences, including "majority vote" [21] and statistical models such as Markov Random field [24]. These methods can assign function based on the network-context of unannotated genes, *i.e*. the number of neighbors that are associated with proteins annotated to a particular category using one or another ontology.

Predictive reliability can be increased by combining them using one or another statistical framework [25,26]such as support vector machines, Bayesian inferences [27] and Markov Random field [17,28], though generally with some loss in predictive coverage. For example, *Y2H *data, which in itself is binary and un-weighted, has been weighted with expression data, and inferences were made using Markov Random field [17]. Context methods work well when properties are highly correlated with those of several other nodes, but effectiveness deteriorates rapidly as correlation stringency drops. As discussed below, *CE *has the desirable property of having relatively good performance even at weak correlations, thus increasing coverage.

Pair-wise protein links based on phylogenetic profiling have also been accumulated in databases such as STRING, Prolinks and Phydbac *etc *[29-32]. The importance of these results is that they are based on a combination of methods, rather than just a single method. However, they all core pairwise links; i.e. they use SGA as a decision rule for individual methods, rather than of gene-category association (CE). Although combining score is important, a combined score is also limited by the decision rule for the individual methods. Here we have focused on a decision rule, which can be applied generally, and developed and evaluated it for phylogenetic profiling using three different ontologies.

Finally we note that McDermott [33] showed using *SGA *to assign genes to *GO *categories, that the fraction of genes assigned correctly to at least one category decreases from 0.98 to ~0.10 as functional specificity increases, with coverage fixed at around 40%. At a comparable coverage using *CE*, the fraction correctly assigned to at least one category is 0.95 at the lowest specificity level, and remains above 0.78 at all specificity levels.

### Identifying functional and evolutionary modules

Several methods have been proposed to identify functional modules [20,34-41]. Here we illustrate module identification by phylogenetic profiling where no specific clustering algorithms are needed.

#### Inferences based on the COG Ontology

*COG *functional categories provide only a low resolution, but fully resolved, annotation. Because the ontology is a one gene to one functional category map, performance assessment is relatively direct; in particular, *A*_*c *_(the average fraction of correct assignments for genes assigned correctly to at least one functional category, eq 7) is 0 or 1, and therefore *PPV *= *A*_0 _(eq 8), the fraction of genes assigned correctly to at least one functional category.

**Figure 3:**
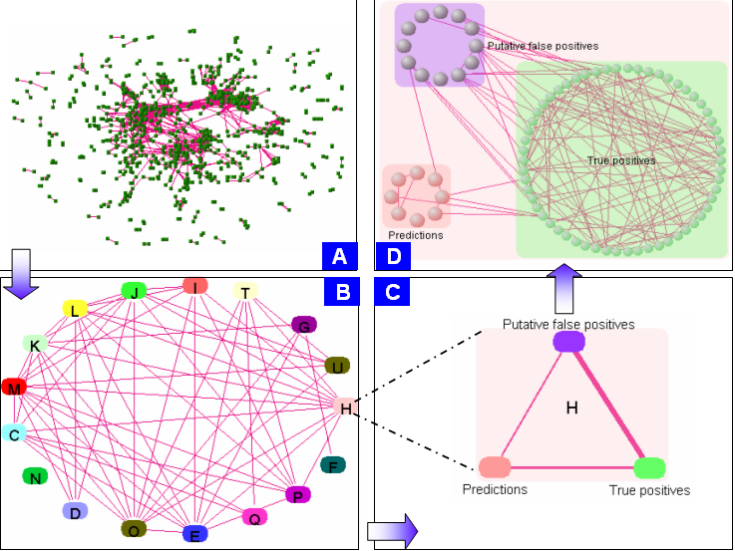
An all against all *VisANT * screen shot, at C* = 0.55, of the 4286 orthologs in the *COG *database. 926 genes (677 annotated; 249 unannotated) are linked to at least one annotated gene. Each gene is unambiguously assigned to a unique *COG *functional category. Of the 677 annotated genes, 463 are correctly assigned; In total 1843 out of 4286 orthologs are unannotated in the *COG *classification. (A) Complete 926 gene network. (B) meta-network of genes from (A). Each group represents a set of genes allocated to a *COG *functional category using *CE*. (C) Detail of functional category *H*, coenzyme metabolism. (D). Of the 926 linked genes, 82 are in category *H*. 62 of them are true positives (green) and 8 are predictions (red). The remaining 12 are annotated in a different functional category and are therefore putative false positives. The minimum *PPV *for category *H *is therefore 62/74 = 0.84 the averaged *PPV *for all categories is estimated to be 68% from all annotated genes. Refer to the *COG *web site for definitions of categories.

An all against all profiling by *CE *of the full set of 4,826 genes, annotated and unannotated, at a threshold of C^* ^= 0.55, returns a 926 genes linked to at least one annotated gene (Figure 3A). Each of the 926 genes, including 249 that are unannotated, is therefore assignable to a *COG *category. Performance is estimated by the fraction of annotated genes that are correctly assigned, which is 68% (463/677).

Sets of genes assigned to the same *COG *functional categories (Figure 3B) are grouped together into meta-nodes (Figure 3C), each containing genes that are classifiable as true or false positives (for annotated genes), or predictions (for unannotated genes). For example, of the 82 genes allocated to category *H *(coenzyme metabolism), 62 of 74 are annotated in category *H *(*PPV *= 0.84), and eight others are predictions. Predictions based on *COG *functional categories can be accessed online [42].

**Figure 4:**
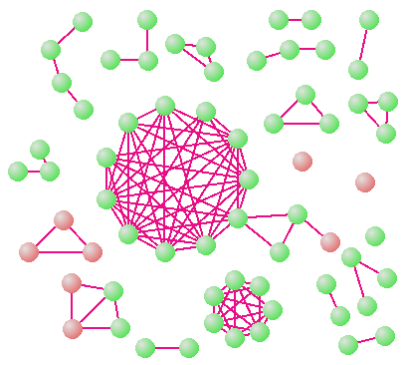
Expanded view of true positive and predicted clusters (Figure 4) in functional category *H*, showing two strikingly dense clusters of size 11 and 7. The elements in the larger cluster all participate in *Porphyrin and chlorophyll metabolism *(*KEGG: *00860), which is a subset of category *H *(coenzyme metabolism).

**Figure 5:**
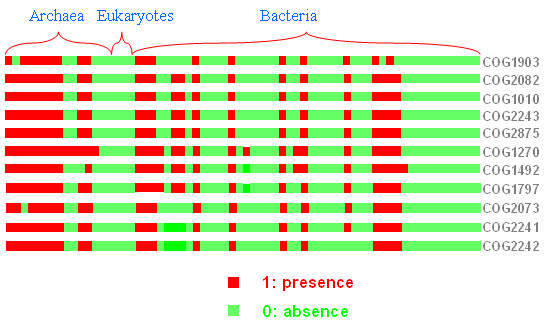
Phylogenetic Profiles of the 11-member cluster (Figure 5) of orthologs across 66 genomes uncovered by *CE*. Green represents absence and red, presence of an ortholog.

A more detailed version of the category *H TP *set (Figure 4) reveals two strikingly dense clusters – one with 7 orthologs, the other with 11. All genes in the latter participate in the P *orphyrin and chlorophyll metabolism *pathway (00860). The cluster is a highly interdependent functional module and it is also strikingly conserved as demonstrated by its aligned profiles (Figure 5). The genes in the seven member cluster are not annotated in *KEGG*. However, four of them are annotated in *GO *and they all share *GO *category 0006777: *molybdopterin cofactor biosynthesis*, at depth 8. It therefore appears likely that the remaining 3 *COG*s are important components of molybdopterin cofactor biosynthesis in one or more genomes. These results indicate the power of *CE *to uncover evolutionarily conserved highly specific functional modules, and to reliably assign previously unannotated genes to these modules.

#### Cliques, clusters and inference quality

Functional modules can be most easily identified by setting a high correlation threshold, discarding all genes that do not meet it, and displaying, as linked nodes, all pairs that exceed the threshold. At the high thresholds used in such an approach, there is no distinction between *CE *and *SGA *for function prediction (See Figure 1).

**Figure 6:**
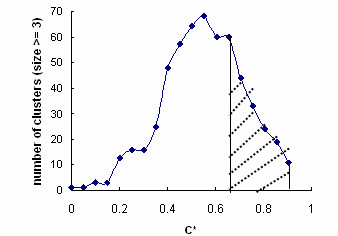
Number of clusters (size >= 3) as a function of C^*^. Shaded area, *i.e*. C^*^>0.7 where *SGA *and *CE *have relatively small PPV difference, is used to extract functional and evolutionary modules.

In general (Figure 6) we find that as the threshold decreases from its most stringent value, (C* = 0.91; p^* ^= 10^-18^) the number of clusters containing more than 3 nodes increases, peaking at C^* ^= 0.66 (p^* ^= 10^-13^) and then declines as the nodes coalesce into increasingly larger clusters. The following remarks are relevant to the region to the right of the peak in figure 6.

**Figure 7:**
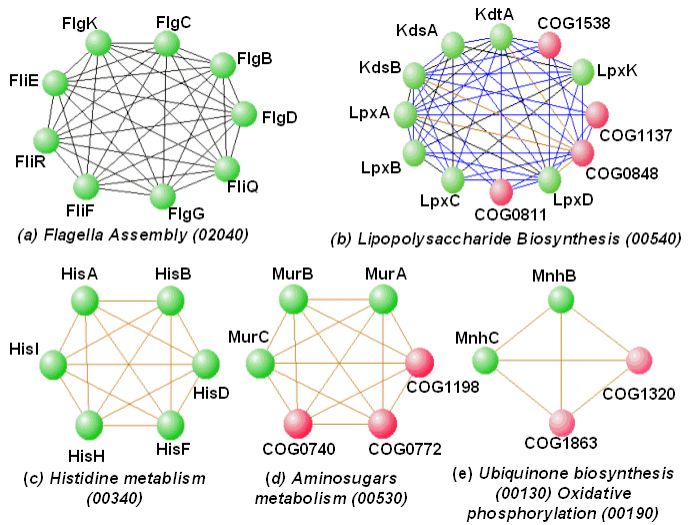
Evolutionarily conserved densely connected clusters. Edge coding: black, C* = 0.91 (p^* ^= 10^-18^); yellow, C* = 0.81 (p^* ^= 10^-16^); blue, C* = 0.71 (p^* ^= 10^-14^). Green nodes correctly annotated; red nodes unannotated. (a) One of 4 cliques uncovered at C* = 0.91. All genes are known and are in the flagella assembly pathway. (b) One of 9 clusters at C* = 0.81, ranging in size from 4 to 13 nodes. In the cluster shown, all genes are annotated to the *KEGG *histidine metabolism pathway and to the *COG *amino acid metabolism and transport category. (c) – (e) are examples of mixed annotated-unannotated clusters, with the annotated sets homogeneous in function. Lower bounds on *PPV *for assigned functions are 79% (C* = 0.71) and 89% (C* = 0.81).

Figure 7 shows examples of five clusters, four of them with clustering coefficients (fraction of pairs that are linked) of 1, and the fifth (the lipopolysaccharide biosynthesis pathway) with a clustering coefficient of 0.875. As we discuss elsewhere such tightly coupled subnets are good candidates for co-regulated sets of genes.

At C* = 0.91 we recover a tightly correlated 9-component fully annotated subnet of the flagella assembly pathway (Figure 7a). In contrast, the 12-node module (Figure 7b), is not fully annotated – but eight of its members are in the *KEGG *lipopolysaccharide biosynthesis pathway. Since it is highly connected (clustering coefficient 0.875), with all linkage strengths equal to or greater than C^* ^= 0.71, enrichment for lipopolysaccharide metabolism is very strong, and each of the unknown *COG*s is almost certainly associated with that function. A weak enrichment-based lower bound on *PPV *is 0.85.

Of the three cliques (c) – (e) one is fully annotated and two are mixed. The former demonstrates recovery of a tightly correlated segment of the histadine metabolism pathway. The latter two are enriched with components of, respectively, the amino sugars metabolism pathway and the ubiquinone biosynthesis pathway. Since they are obtained at C^* ^= 0.81, the unannotated genes are likely to be in the indicated pathway, a conservative estimate of accuracy of assignments (from eq 5) being 94%

More generally for C* = 0.71, there are 20 cliques and quasi-cliques. Of these, 10 are partially annotated. Their properties, and lower bounds on the correct allocation of the unknown orthologs to the majority function of the cliques, are available online [43]. Similar remarks hold for the six node clique, which has four genes implicated in the aminosugars metabolism pathway.

Four genes in the smallest clique are part of a multi-subunit complex, which is has a descriptor Na^+ ^/H^+ ^antiporter in the COG ontology. Two of the domains have *KEGG *annotations in the *ubiquinone biosynthesis *pathway (00130) and *oxidative phosphorylation *pathway (00190) in a subset of the genomes in which they co-occur. In the other genomes in which they co-occur, pathway annotation is missing. The strong correlation obtained between these two annotated domains is plausible since ubiquinone is known to be involved in respiratory chain oxidative phosphorylation. In addition, all links (annotated-annotated, annotated-unannotated, and unannotated-annotated) are equally strong, suggesting that the two unannotated genes are also required for the respiratory chain in the genomes in which the other two are annotated, including *Pyrococcus horikoshii, Pyrococcus abyssi*, and *Rickettsia prowazekii*.

Our predictions not only suggest functions for unannotated genes but also add new functions for annotated genes. These plausible functions do not contradict the existing annotation but rather, amplify a pleiotropic theme *i.e*. proteins can have multiple functions. In fact, on average each gene is assigned to 2.79 pathways in *KEGG *and 2–3 *GO *categories at all levels. Even genes clustered at the most stringent C^* ^threshold (Figure 7a) are assigned to more than one pathways, *e.g. fliQ *is not only assigned to flagella assembly pathway (02040) but also to Type III secretion system (03070).

## Conclusion

The method serves as a general computational tool for annotating large numbers of unknown genes, uncovering evolutionary and functional modules. It appears to perform substantially better than extant stand alone high throughout methods.

Finally, we note that a potentially fundamental limitation of phylogenetic profiling is the confounding influence of correlations between genomes, as opposed to correlations between genes. While we do not report a complete study of the effect of inter-genome correlations, we estimated its potential influence by collapsing those genomes that are phylogenetically close, essentially assuming that all correlations between gene pairs that are present within a group of related genomes are the result of genome correlation rather than gene correlation. We find that, for this conservative model, genome correlations have only a small effect on the performance of the method given a reasonable number of lineages. In fact, when 66 genomes are collapsed (*i.e*. closely related species are represented by a single digit in profiles) to as few as 32 lineages based on their phylogenetic distances measured by genome content [44], the corresponding change in *PPV *for the same coverage is always less than 1%.

We conclude that a principal source of variance between phylogenetic correlation and category assignments is in the way proteins are grouped by the ontologies. A given level of functional correlation between genes, as determined by any particular correlate, whether experimental (the 2-hybrid assay) or computational, does not assure a particular level of category specificity (*e.g*. presence in the same pathway), nor does co-presence at a particular category specificity level assure that a given level of correlation will be achieved. Representing relations between genes in accordance with ontological categories on the one hand or in accordance with evolutionary or biochemical correlations on the other, have elements of arbitrariness and uncertainty and consequently are expected to yield, to the extent that they are valid, overlapping but not identical classifications.

## Methods

### The dataset

We adhere to the conventions of the *COG *database [10] and construct profiles only for genes that occur in at least three lineages. All paralogs are collapsed; *i.e*. a set of closely related genes in a given lineage is treated as a single entity. The analysis was performed for 4,873 clusters of orthologs (*COGs*) from 66 fully sequenced microbial genomes in the three domains of life. Accuracy is evaluated against the *Kyoto Encyclopedia of Genes and Genomes *[45]; the *Gene Ontology Consortium *(2000) [46]; 23 *COG *broad functional categories; annotations for 6059 *Saccharomyces cerevisiae *ORFs (*SGD *[47]) and 4410 *E.coli K12 *ORFs (*EcoCyc *[48]). Of the 206 biochemical pathways in *KEGG*, we used 133 (mostly metabolic pathways); in particular those that are generic. These pathways contain a total of 1,368 orthologs.

### Assessment

Eq 3b is used to assign unannotated genes to *KEGG*, *GO *or *COG *categories using a *Guilt by association *or *Correlation enrichment *decision rule. For each gene we assess true positives (*TP*), *i.e*. the number of categories correctly assigned; false positives (*FP*), the number of categories incorrectly assigned; true negatives (*TN*), the number categories to which it is not assigned, and in which it is not annotated; false negatives (*FN*) the number of categories to which it is not assigned and in which it is annotated. The sensitivity (*SEN*), specificity (*SPE*), accuracy (*AC*C) and positive predictive value (*PPV*) are functions of these four quantities

SEN=TP/(TP+FN)(4a)SPE=TN/(TN+FP)(4b)ACC=(TP+TN)/(TP+TN+FP+FN)(4c)PPV=TP/(TP+FP)(4d)     (4)
 MathType@MTEF@5@5@+=feaafiart1ev1aaatCvAUfKttLearuWrP9MDH5MBPbIqV92AaeXatLxBI9gBaebbnrfifHhDYfgasaacH8akY=wiFfYdH8Gipec8Eeeu0xXdbba9frFj0=OqFfea0dXdd9vqai=hGuQ8kuc9pgc9s8qqaq=dirpe0xb9q8qiLsFr0=vr0=vr0dc8meaabaqaciaacaGaaeqabaqabeGadaaakeaafaqaaeabcaaaaeaacqWGtbWucqWGfbqrcqWGobGtcqGH9aqpcqWGubavcqWGqbaucqGGVaWlcqGGOaakcqWGubavcqWGqbaucqGHRaWkcqWGgbGrcqWGobGtcqGGPaqkaeaacqGGOaakcqqG0aancqqGHbqycqGGPaqkaeaacqWGtbWucqWGqbaucqWGfbqrcqGH9aqpcqWGubavcqWGobGtcqGGVaWlcqGGOaakcqWGubavcqWGobGtcqGHRaWkcqWGgbGrcqWGqbaucqGGPaqkaeaacqGGOaakcqqG0aancqqGIbGycqGGPaqkaeaacqWGbbqqcqWGdbWqcqWGdbWqcqGH9aqpcqGGOaakcqWGubavcqWGqbaucqGHRaWkcqWGubavcqWGobGtcqGGPaqkcqGGVaWlcqGGOaakcqWGubavcqWGqbaucqGHRaWkcqWGubavcqWGobGtcqGHRaWkcqWGgbGrcqWGqbaucqGHRaWkcqWGgbGrcqWGobGtcqGGPaqkaeaacqGGOaakcqqG0aancqqGJbWycqGGPaqkaeaacqWGqbaucqWGqbaucqWGwbGvcqGH9aqpcqWGubavcqWGqbaucqGGVaWlcqGGOaakcqWGubavcqWGqbaucqGHRaWkcqWGgbGrcqWGqbaucqGGPaqkaeaacqGGOaakcqqG0aancqqGKbazcqGGPaqkaaGaaCzcaiaaxMaadaqadaqaaiabisda0aGaayjkaiaawMcaaaaa@86B4@

#### Positive Predictive Value

The quantity of natural interest for assessing threshold based predictions is *PPV *(or *precision*). By definition, the population averaged positive predictive value is

PPV¯=1Na∑I=1NaPPVI     (5)
 MathType@MTEF@5@5@+=feaafiart1ev1aaatCvAUfKttLearuWrP9MDH5MBPbIqV92AaeXatLxBI9gBaebbnrfifHhDYfgasaacH8akY=wiFfYdH8Gipec8Eeeu0xXdbba9frFj0=OqFfea0dXdd9vqai=hGuQ8kuc9pgc9s8qqaq=dirpe0xb9q8qiLsFr0=vr0=vr0dc8meaabaqaciaacaGaaeqabaqabeGadaaakeaadaqdaaqaaiabdcfaqjabdcfaqjabdAfawbaacqGH9aqpdaWcaaqaaiabigdaXaqaaiabd6eaonaaBaaaleaacqWGHbqyaeqaaaaakmaaqahabaGaemiuaaLaemiuaaLaemOvay1aaSbaaSqaaiabdMeajbqabaaabaGaemysaKKaeyypa0JaeGymaedabaGaemOta40aaSbaaWqaaiabdggaHbqabaaaniabggHiLdGccaWLjaGaaCzcamaabmaabaGaeGynaudacaGLOaGaayzkaaaaaa@4572@

where *N*_*a *_is the total number of annotated genes linked at C* and *PPV*_*I*_, the positive predictive value for unannotated gene *I*, is given by eq 4d. Analogous equations hold for the other measures of performance.

Comparison with results in the literature is facilitated by writing PPV¯
 MathType@MTEF@5@5@+=feaafiart1ev1aaatCvAUfKttLearuWrP9MDH5MBPbIqV92AaeXatLxBI9gBaebbnrfifHhDYfgasaacH8akY=wiFfYdH8Gipec8Eeeu0xXdbba9frFj0=OqFfea0dXdd9vqai=hGuQ8kuc9pgc9s8qqaq=dirpe0xb9q8qiLsFr0=vr0=vr0dc8meaabaqaciaacaGaaeqabaqabeGadaaakeaadaqdaaqaaiabdcfaqjabdcfaqjabdAfawbaaaaa@3044@ as a product of two factors: the fraction of genes that are correctly assigned to at least one functional category (*A*_0_), and the average fraction of those assignments that are correct (*A*_*C*_).

Let *N*_*c *_be the number of genes that are assigned correctly to at least one functional category. Then

A0=NcNa     (6)
 MathType@MTEF@5@5@+=feaafiart1ev1aaatCvAUfKttLearuWrP9MDH5MBPbIqV92AaeXatLxBI9gBaebbnrfifHhDYfgasaacH8akY=wiFfYdH8Gipec8Eeeu0xXdbba9frFj0=OqFfea0dXdd9vqai=hGuQ8kuc9pgc9s8qqaq=dirpe0xb9q8qiLsFr0=vr0=vr0dc8meaabaqaciaacaGaaeqabaqabeGadaaakeaacqWGbbqqdaWgaaWcbaGaeGimaadabeaakiabg2da9maalaaabaGaemOta40aaSbaaSqaaiabdogaJbqabaaakeaacqWGobGtdaWgaaWcbaGaemyyaegabeaaaaGccaWLjaGaaCzcamaabmaabaGaeGOnaydacaGLOaGaayzkaaaaaa@3908@

Ac=1Nc∑I=1NcTPITPI+FPI=1Nc∑I=1NcPPVI     (7)
 MathType@MTEF@5@5@+=feaafiart1ev1aaatCvAUfKttLearuWrP9MDH5MBPbIqV92AaeXatLxBI9gBaebbnrfifHhDYfgasaacH8akY=wiFfYdH8Gipec8Eeeu0xXdbba9frFj0=OqFfea0dXdd9vqai=hGuQ8kuc9pgc9s8qqaq=dirpe0xb9q8qiLsFr0=vr0=vr0dc8meaabaqaciaacaGaaeqabaqabeGadaaakeaacqWGbbqqdaWgaaWcbaGaem4yamgabeaakiabg2da9maalaaabaGaeGymaedabaGaemOta40aaSbaaSqaaiabdogaJbqabaaaaOWaaabCaeaadaWcaaqaaiabdsfaujabdcfaqnaaBaaaleaacqWGjbqsaeqaaaGcbaGaemivaqLaemiuaa1aaSbaaSqaaiabdMeajbqabaGccqGHRaWkcqWGgbGrcqWGqbaudaWgaaWcbaGaemysaKeabeaaaaaabaGaemysaKKaeyypa0JaeGymaedabaGaemOta40aaSbaaWqaaiabdogaJbqabaaaniabggHiLdGccqGH9aqpdaWcaaqaaiabigdaXaqaaiabd6eaonaaBaaaleaacqWGJbWyaeqaaaaakmaaqahabaGaemiuaaLaemiuaaLaemOvay1aaSbaaSqaaiabdMeajbqabaaabaGaemysaKKaeyypa0JaeGymaedabaGaemOta40aaSbaaWqaaiabdogaJbqabaaaniabggHiLdGccaWLjaGaaCzcamaabmaabaGaeG4naCdacaGLOaGaayzkaaaaaa@5CE6@

and the population averaged positive predictive value is

PPV¯
 MathType@MTEF@5@5@+=feaafiart1ev1aaatCvAUfKttLearuWrP9MDH5MBPbIqV92AaeXatLxBI9gBaebbnrfifHhDYfgasaacH8akY=wiFfYdH8Gipec8Eeeu0xXdbba9frFj0=OqFfea0dXdd9vqai=hGuQ8kuc9pgc9s8qqaq=dirpe0xb9q8qiLsFr0=vr0=vr0dc8meaabaqaciaacaGaaeqabaqabeGadaaakeaadaqdaaqaaiabdcfaqjabdcfaqjabdAfawbaaaaa@3044@ = *A*_0_*A*_*C *_    (8)

*i.e*. *A*_*C *_is the average positive predictive value of genes that are assigned correctly at least once, and *A*_0 _is the fraction of annotated genes assigned correctly at least once. Although *A*_0 _is sometimes used as a measure of PPV¯
 MathType@MTEF@5@5@+=feaafiart1ev1aaatCvAUfKttLearuWrP9MDH5MBPbIqV92AaeXatLxBI9gBaebbnrfifHhDYfgasaacH8akY=wiFfYdH8Gipec8Eeeu0xXdbba9frFj0=OqFfea0dXdd9vqai=hGuQ8kuc9pgc9s8qqaq=dirpe0xb9q8qiLsFr0=vr0=vr0dc8meaabaqaciaacaGaaeqabaqabeGadaaakeaadaqdaaqaaiabdcfaqjabdcfaqjabdAfawbaaaaa@3044@ (and sometimes referred to as accuracy ([3,4,21,33]), in general, *A*_0 _is a very poor measure of PPV¯
 MathType@MTEF@5@5@+=feaafiart1ev1aaatCvAUfKttLearuWrP9MDH5MBPbIqV92AaeXatLxBI9gBaebbnrfifHhDYfgasaacH8akY=wiFfYdH8Gipec8Eeeu0xXdbba9frFj0=OqFfea0dXdd9vqai=hGuQ8kuc9pgc9s8qqaq=dirpe0xb9q8qiLsFr0=vr0=vr0dc8meaabaqaciaacaGaaeqabaqabeGadaaakeaadaqdaaqaaiabdcfaqjabdcfaqjabdAfawbaaaaa@3044@ and provides an overly optimistic assessment of performance.

#### Related metrics

##### SPE-ACC

For category allocation, specificity and accuracy will be quantitatively very similar; *i.e*. true negatives will invariably be much greater than true positives and false negatives, owing to the fact that the vast majority of genes are in a small fraction of all pathways. Consequently we expect *SPE *≈ *ACC*.

##### SEN-*A*_0_

Whereas SPE and ACC are quantitatively similar, SEN and the fraction of genes that are correctly allocated at least once (A_0_) are qualitatively similar. At very high coverage, the threshold is so weak that almost every gene is linked correctly at least once (A_0 _and sensitivity are high); at low coverage the threshold is so stringent that true positives are greater than FN, and again A_0 _and sensitivity are high. In short, A_0 _is similar to sensitivity and slightly larger than sensitivity at all coverages. The similarity is strong enough so that they provide the same measure of performance.

Hence of the 5 measures, only three are independent. These are traditionally taken as SEN, SPE and PPV. (A fourth measure, negative predictive value, which adds little to the discussion, is omitted in the interest of brevity). Performance is measured by their functional dependence on coverage. These definitions are introduced in terms of a particular gene. Passing to population averaged quantities is in principle direct, although in practice it involves some care because of cross correlations between categories.

The final quantity of interest is coverage, defined as the fraction of genes (unannotated or annotated) that can be linked to at least one annotated gene.

Of the various measures of performance, the two most informative for the decision rules of interest here are *PPV *and *SPE*. Sensitivity, for example, is not informative because it is high at both high and low coverage (at very high coverage, the threshold is so weak that almost every gene is linked correctly at least once; at low coverage the threshold is so stringent that true positives are greater than *FN*, and again *SEN *is very high.). The same is true for the related quantity, *A*_0_. We therefore focus on positive predictive value and specificity as a function of coverage. We compare decision rules, first generically on the basis of ability to allocate orthologs to *KEGG *pathways, and then for specific genomes; in particular, yeast and *E. Coli*.

### Standard guilt by association (SGA)

An unannotated gene generally meets the threshold condition

*C*(*z *| *N*, *x*, *y*) ≥ C*

with multiple genes, and each associated gene typically participates in more than one process. The unannotated gene is of necessity assigned to all categories of the gene to which it is linked.

In order to develop performance measures, let *i *be the number of the categories that contain the *gene I*, whose biological function is to be predicted; let *J*(*I*, *J*) be the set of categories that contain a gene *J *whose profile correlation with *I *meets the threshold C*, *j*(*I*, *J*) is its size, and let *K*(*I*, *J*) denote the set of common categories and *k*(*I*, *J*) is its size; where 0 ≤ *k*(*I*, *J*) ≤ min(*i*, *j*). The unannotated gene is therefore correctly assigned to *TP *= *k *categories, and incorrectly assigned to the remaining *FP *= *j *- *k *categories. Also *TN *= *T *- *i *- *j *+ *k *and *FN *= *i *- *k*, where *T *= 133 is the total number of pathways. Consequently, the *PPV*_*I*_(*J*) with which gene *I *is assigned using linked gene *J *is

PPVI(J)=kj     (9)
 MathType@MTEF@5@5@+=feaafiart1ev1aaatCvAUfKttLearuWrP9MDH5MBPbIqV92AaeXatLxBI9gBaebbnrfifHhDYfgasaacH8akY=wiFfYdH8Gipec8Eeeu0xXdbba9frFj0=OqFfea0dXdd9vqai=hGuQ8kuc9pgc9s8qqaq=dirpe0xb9q8qiLsFr0=vr0=vr0dc8meaabaqaciaacaGaaeqabaqabeGadaaakeaacqWGqbaucqWGqbaucqWGwbGvdaWgaaWcbaGaemysaKeabeaakiabcIcaOiabdQeakjabcMcaPiabg2da9maalaaabaGaem4AaSgabaGaemOAaOgaaiaaxMaacaWLjaWaaeWaaeaacqaI5aqoaiaawIcacaGLPaaaaaa@3BF2@

Note that the maximum *PPV*_*I*_(*J*) is not necessarily 1, but min(*i*, *j*)/*j*.

For *j *>*i*, *PPV*_*I *_< 1, whereas when *i > j*, *PPV*_*I*_(*J*) can become 1 when the pathways of *J *are a subset of those of *I*. The positive predictive value for gene *I *is obtained by taking sums over all genes to which it is correlated.

PPVI=∪J=1G(I)K(I,J)∪J=1G(I)J(I,J)=∪J=1Nc(I)K(I,J)∪J=1G(I)J(I,J)     (10)
 MathType@MTEF@5@5@+=feaafiart1ev1aaatCvAUfKttLearuWrP9MDH5MBPbIqV92AaeXatLxBI9gBaebbnrfifHhDYfgasaacH8akY=wiFfYdH8Gipec8Eeeu0xXdbba9frFj0=OqFfea0dXdd9vqai=hGuQ8kuc9pgc9s8qqaq=dirpe0xb9q8qiLsFr0=vr0=vr0dc8meaabaqaciaacaGaaeqabaqabeGadaaakeaacqWGqbaucqWGqbaucqWGwbGvdaWgaaWcbaGaemysaKeabeaakiabg2da9maalaaabaWaambCaeaacqWGlbWscqGGOaakcqWGjbqscqGGSaalcqWGkbGscqGGPaqkaSqaaiabdQeakjabg2da9iabigdaXaqaaiabdEeahjabcIcaOiabdMeajjabcMcaPaqdcqWIQisvaaGcbaWaambCaeaacqWGkbGscqGGOaakcqWGjbqscqGGSaalcqWGkbGscqGGPaqkaSqaaiabdQeakjabg2da9iabigdaXaqaaiabdEeahjabcIcaOiabdMeajjabcMcaPaqdcqWIQisvaaaakiabg2da9maalaaabaWaambCaeaacqWGlbWscqGGOaakcqWGjbqscqGGSaalcqWGkbGscqGGPaqkaSqaaiabdQeakjabg2da9iabigdaXaqaaiabd6eaonaaBaaameaacqWGJbWyaeqaaSGaeiikaGIaemysaKKaeiykaKcaniablQIivbaakeaadaWeWbqaaiabdQeakjabcIcaOiabdMeajjabcYcaSiabdQeakjabcMcaPaWcbaGaemOsaOKaeyypa0JaeGymaedabaGaem4raCKaeiikaGIaemysaKKaeiykaKcaniablQIivbaaaOGaaCzcaiaaxMaadaqadaqaaiabigdaXiabicdaWaGaayjkaiaawMcaaaaa@74E0@

where *G*(*I*) is the number of genes correlated with gene *I *and *N*_*c*_(*I*) is the subset of genes in *G*(*I*) that share at least one category with gene *I*, *i.e*. fraction of assigned categories that are correct. Here union symbol is used instead of a sum to indicate avoidance of double counting when a category has more than a single gene linked to the unannotated gene. *A*_*c *_is given by substituting eq 10 into eq 7.

### Correlation enrichment (CE)

Suppose an unannotated gene is correlated with in total *g *other genes (*C *>C^*^) from *r *categories, and let *m*_1_, *m*_2_, ..., *m*_*r *_be the number of correlated genes in categories *k*_1_, *k*_2_, ..., *k*_*r*_, where *r *≤ *g*, the equality holding only when each gene is in one category. Further, let k′1,k′1,…,k′TI
 MathType@MTEF@5@5@+=feaafiart1ev1aaatCvAUfKttLearuWrP9MDH5MBPbIqV92AaeXatLxBI9gBaebbnrfifHhDYfgasaacH8akY=wiFfYdH8Gipec8Eeeu0xXdbba9frFj0=OqFfea0dXdd9vqai=hGuQ8kuc9pgc9s8qqaq=dirpe0xb9q8qiLsFr0=vr0=vr0dc8meaabaqaciaacaGaaeqabaqabeGadaaakeaacuWGRbWAgaqbamaaBaaaleaacqaIXaqmaeqaaOGaeiilaWIafm4AaSMbauaadaWgaaWcbaGaeGymaedabeaakiabcYcaSiablAciljabcYcaSiqbdUgaRzaafaWaaSbaaSqaaiabdsfaunaaBaaameaacqWGjbqsaeqaaaWcbeaaaaa@39AB@ denote the categories the gene is in. For each of the *r *categories that have 1 or more genes meeting the correlation threshold with *I*, define a weighted sum score, *S*_*v*_

Sv=∑j=1mv[−log⁡P]αv=1…r     (11)
 MathType@MTEF@5@5@+=feaafiart1ev1aaatCvAUfKttLearuWrP9MDH5MBPbIqV92AaeXatLxBI9gBaebbnrfifHhDYfgasaacH8akY=wiFfYdH8Gipec8Eeeu0xXdbba9frFj0=OqFfea0dXdd9vqai=hGuQ8kuc9pgc9s8qqaq=dirpe0xb9q8qiLsFr0=vr0=vr0dc8meaabaqaciaacaGaaeqabaqabeGadaaakeaafaqabeqacaaabaGaem4uam1aaSbaaSqaaGqaciab=zha2bqabaGccqGH9aqpdaaeWbqaaiabcUfaBjabgkHiTiGbcYgaSjabc+gaVjabcEgaNjabdcfaqjabc2faDnaaCaaaleqabaacciGae4xSdegaaaqaaiabdQgaQjabg2da9iabigdaXaqaaiabd2gaTnaaBaaameaacqWF2bGDaeqaaaqdcqGHris5aaGcbaGaemODayNaeyypa0JaeGymaeJaeSOjGSKaemOCaihaaiaaxMaacaWLjaWaaeWaaeaacqaIXaqmcqaIXaqmaiaawIcacaGLPaaaaaa@4E62@*v *= 1 ... *r *    (11)

*α *is a positive adjustable integer which gives disproportionately high weights to strong correlations. Thus a linked pathway is weighted by a combination of the number of genes in the pathway, which exceed the threshold, and the phylogenetic profile similarity of those genes to the one being tested. Un-weighted ranking, in which only the number of genes is used, is a special case with *α *= 0. Tests using different *α *indicate that *α *= 4 is optimal. *P *is calculated from equation (1) using the profile of the gene and those of genes in the category under consideration. The category scores *S*_*v *_are ranked in descending order and the unnnotated gene is allocated to the top *r*_0 _categories. The number of true positives is the intersection between the categories the unannotated gene is in (*T*_*I*_), and these *r*_0 _categories. Then *FP *= *r*_0 _-*TP*, *FN *= *T*_*I *_- *TP*, *TN *= *T *- *r*_0 _- *T*_*I *_+ *TP *

PPVI=TPTP+FP=∑j=1TI∑v=1r0δ(kv−k′j)r0     (12)
 MathType@MTEF@5@5@+=feaafiart1ev1aaatCvAUfKttLearuWrP9MDH5MBPbIqV92AaeXatLxBI9gBaebbnrfifHhDYfgasaacH8akY=wiFfYdH8Gipec8Eeeu0xXdbba9frFj0=OqFfea0dXdd9vqai=hGuQ8kuc9pgc9s8qqaq=dirpe0xb9q8qiLsFr0=vr0=vr0dc8meaabaqaciaacaGaaeqabaqabeGadaaakeaacqWGqbaucqWGqbaucqWGwbGvdaWgaaWcbaGaemysaKeabeaakiabg2da9maalaaabaGaemivaqLaemiuaafabaGaemivaqLaemiuaaLaey4kaSIaemOrayKaemiuaafaaiabg2da9maaqahabaWaaabCaeaadaWcaaqaaGGaciab=r7aKjabcIcaOiabdUgaRnaaBaaaleaacqWG2bGDaeqaaOGaeyOeI0Iafm4AaSMbauaadaWgaaWcbaGaemOAaOgabeaakiabcMcaPaqaaiabdkhaYnaaBaaaleaacqaIWaamaeqaaaaaaeaaieGacqGF2bGDcqGH9aqpcqaIXaqmaeaacqWGYbGCdaWgaaadbaGaeGimaadabeaaa0GaeyyeIuoaaSqaaiabdQgaQjabg2da9iabigdaXaqaaiabdsfaunaaBaaameaacqWGjbqsaeqaaaqdcqGHris5aOGaaCzcaiaaxMaadaqadaqaaiabigdaXiabikdaYaGaayjkaiaawMcaaaaa@5D55@

where δ(j−v)={0,kv≠k′j1,kv=k′jand 0≤PPVI≤1
 MathType@MTEF@5@5@+=feaafiart1ev1aaatCvAUfKttLearuWrP9MDH5MBPbIqV92AaeXatLxBI9gBaebbnrfifHhDYfgasaacH8akY=wiFfYdH8Gipec8Eeeu0xXdbba9frFj0=OqFfea0dXdd9vqai=hGuQ8kuc9pgc9s8qqaq=dirpe0xb9q8qiLsFr0=vr0=vr0dc8meaabaqaciaacaGaaeqabaqabeGadaaakeaafaqabeqacaaabaqbaeqabeGaaaqaaGGaciab=r7aKjabcIcaOiabdQgaQjabgkHiTiabdAha2jabcMcaPiabg2da9aqaaiabcUha7vaabeqaciaaaeaacqaIWaamcqGGSaalaeaacqWGRbWAdaWgaaWcbaGaemODayhabeaakiabgcMi5kqbdUgaRzaafaWaaSbaaSqaaiabdQgaQbqabaaakeaacqaIXaqmcqGGSaalaeaacqWGRbWAdaWgaaWcbaGaemODayhabeaakiabg2da9iqbdUgaRzaafaWaaSbaaSqaaiabdQgaQbqabaaaaaaaaOqaaiabbggaHjabb6gaUjabbsgaKjabbccaGiabicdaWiabgsMiJkabdcfaqjabdcfaqjabdAfawnaaBaaaleaacqWGjbqsaeqaaOGaeyizImQaeGymaedaaaaa@57D4@

An analysis of *KEGG *and *GO *indicates that the average number of functional categories per gene is between 2 and 3. It would therefore seem reasonable to take *r*_0 _= 3 for *KEGG *and *GO*, where a relatively large number of categories is available; *i.e*. we allocate to at most 3 categories. We use the more stringent condition *r*_0 _= 1, for the relatively coarse grained *COG *ontology. For *COG *categories, *PPV*_*I *_= 1 or 0,

*PPV *= *A*_0 _= *N*_*c *_/*N*_*a*_.     (13)

## List of abbreviations

**SGA: **Standard Guilt by Association

**CE: **Correlation Enrichment

**GO: **Gene Ontology

**KEGG: **Kyoto Encyclopedia of Genes and Genomes

**COG: **Clusters of orthologous groups

**PPV: **Positive Predictive Value

**PPI: **Protein-protein interaction
